# Vascular Cognitive Impairment After Mild Stroke: Connectomic Insights, Neuroimaging, and Knowledge Translation

**DOI:** 10.3389/fnins.2022.905979

**Published:** 2022-07-07

**Authors:** Jess A. Holguin, John L. Margetis, Anisha Narayan, Grant M. Yoneoka, Andrei Irimia

**Affiliations:** ^1^T.H. Chan Division of Occupational Science and Occupational Therapy, University of Southern California, Los Angeles, CA, United States; ^2^Tulane University School of Medicine, Tulane University, New Orleans, LA, United States; ^3^John A. Burns School of Medicine, University of Hawai‘i at Mānoa, Honolulu, HI, United States; ^4^Leonard Davis School of Gerontology, Ethel Percy Andrus Gerontology Center, University of Southern California, Los Angeles, CA, United States; ^5^Corwin D. Denney Research Center, Department of Biomedical Engineering, Viterbi School of Engineering, University of Southern California, Los Angeles, CA, United States

**Keywords:** stroke, neuroimaging, occupational therapy, neurorehabilitation, cognitive dysfunction, neurocognitive function, translational medical research, connectomics

## Abstract

Contemporary stroke assessment protocols have a limited ability to detect vascular cognitive impairment (VCI), especially among those with subtle deficits. This lesser-involved categorization, termed mild stroke (MiS), can manifest compromised processing speed that negatively impacts cognition. From a neurorehabilitation perspective, research spanning neuroimaging, neuroinformatics, and cognitive neuroscience supports that processing speed is a valuable proxy for complex neurocognitive operations, insofar as inefficient neural network computation significantly affects daily task performance. This impact is particularly evident when high cognitive loads compromise network efficiency by challenging task speed, complexity, and duration. Screening for VCI using processing speed metrics can be more sensitive and specific. Further, they can inform rehabilitation approaches that enhance patient recovery, clarify the construct of MiS, support clinician-researcher symbiosis, and further clarify the occupational therapy role in targeting functional cognition. To this end, we review relationships between insult-derived connectome alterations and VCI, and discuss novel clinical approaches for identifying disruptions of neural networks and white matter connectivity. Furthermore, we will frame knowledge translation efforts to leverage insights from cutting-edge structural and functional connectomics research. Lastly, we highlight how occupational therapists can provide expertise as knowledge brokers acting within their established scope of practice to drive substantive clinical innovation.

## Introduction

Stroke is the most frequent cause of disability in the United States (Ovbiagele and Nguyen-Huynh, 2011), a fact that spurs investigation into the nature and variability of infarct-related deficits along a continuum of impairment. The literature is replete with widely accepted functional characterizations of moderate through severe stroke (Murphy et al., 2001; Hodics et al., 2012; Rost et al., 2016), with less clarity available on mild clinical presentations (Brott et al., 1989; Roberts et al., 2020). This uncertainty stems from the absence of a precise taxonomy for characterizing the mild stroke (MiS) population (Roberts et al., 2020). In clinical practice, the lack of a consensus definition precludes consistency in evaluation and treatment approaches and obscures understanding of this population’s needs.

Ongoing work to develop an accord on MiS codification can benefit both research and clinical practice. In their systematic review on downstream effects of inconsistent MiS classification, Roberts et al. (2020) discuss 10 distinct definitions present in the literature. This lack of diagnostic and taxonomic uniformity potentiates knowledge translation efforts targeting the depth and breadth of understanding within this important stroke practice and research area. However, even a robust consensus definition cannot explain mechanisms that drive variation in post-stroke disability, especially regarding shared and distinct underpinnings among each NIH Stroke Scale (NIHSS) severity level (National Institute of Neurological Disorders and Stroke, 2011).

Within this review, we address MiS-relevant associations between vascular cognitive impairment (VCI), processing speed (PS), and neural network efficiency, as informed by insights from neuroimaging and connectomics research. We present evidence suggesting that established norms for key neurocognitive assessments can be used as proxies for detecting potentially overlooked VCI. Infusing emerging theoretical perspectives from multiple academic disciplines, we review approaches that can fuel substantial clinical innovation. In particular, we focus on using proxy-defined, threshold-specific instances of dysfunction that are scalable according to premorbid capacities and inherent daily routine demands. Drawing from neuroimaging-informed models employed to predict and monitor stroke recovery, we argue that performance capacity can be quantified by the degree of dissociation between available and necessary performance skills. Such quantification could empower clinicians and MiS survivors to more strategically consider interrelationships between current abilities and requisite progress along an ecologically valid, individualized recovery timeline.

We also examine three intersecting themes that provide a framework for early MiS care innovation and highlight paths to advance clinical investigation targeting health and wellbeing. After providing background on essential considerations of stroke and associated cognitive sequelae, we first review the problem of MiS-associated-VCI (MiS-VCI) underdetection and clarify the consequences of imprecisely characterizing stroke-derived neurocognitive dysfunction. Secondly, we examine the promise of knowledge translation efforts to improve stroke care and address priorities articulated by healthcare systems and research funding agencies. Thirdly, we overview and delineate knowledge relevant to MiS-VCI rehabilitation that derives from cutting-edge neuroanatomic, structural, and functional connectomics research. These studies employ advanced neuroimaging technologies plus conventional computed tomography (CT) and magnetic resonance imaging (MRI). Importantly, we focus on PS as a proxy for the integrity of neural networks and neurocognitive capacities. Lastly, we highlight clinical implications and future directions by providing evidence and arguments supporting more comprehensive MiS-VCI screening in early stroke care and emphasize the pivotal role of occupational therapy (OT) in addressing functional cognition.

While calling attention to the valuable confluence of contemporary research findings and clinical stroke rehabilitation practices, we will discuss literature ranging from the acute to more chronic phases of recovery. Beyond temporal considerations, foundational links between network theory and neurological insults are influenced by a broad range of factors such as demographic characteristics, lesion laterality, and even mechanisms of injury (e.g., ischemic vs. hemorrhagic stroke vs. traumatic brain injury). Herein, we do not focus on such differentiating factors, as it would far exceed the scope of this endeavor and is the likely purview of future prospective data-analytic studies. We do, however, discuss in detail the findings from conceptual and applied perspectives on an overarching construct poised to drive innovation in clinical practice. Please see Table 1. for a summary of constructs and interrelationships.

**TABLE 1:** Summary of overarching constructs, themes, and seminal works within the reviewed sources.

References	Summary Points
**MiS classification and VCI underdetection**	
Roberts et al., 2020	Highlights inconsistencies in MiS classification linked to contemporary methods of assessment, imaging, and outcomes. Discusses 10 different definitions present in the literature.
Fischer et al., 2010	Examined six MiS definitions and outcomes, testing their validity in stroke patient subgroups. Determined that definitions best suited for this population were: (a) those with a score of 1 or less on every NIHSS item and normal consciousness; or (b) an NIHSS score of three or less.
Burns et al., 2018	Presents findings from The Health and Wellness Task Force that emphasize return to work and driving as being key aspects of community reintegration following stroke. Discusses “mild stroke” as a misnomer in light of the persistent challenges experienced by MiS survivors. Notes the disconnect between patients’ needs and available services to address them. Proposes an interdisciplinary MiS practice model to assist those described as frequently being discharged from the hospital without appropriate rehabilitation services targeting complex life activities.
Wolf and Rognstad, 2013	Study with findings indicating “.performance on standardized cognitive testing in the early phases of mild stroke remained stable over a 6-month period. These results help justify the necessity and ability to assess cognition immediately post-mild stroke in order to make accurate and appropriate rehabilitation recommendations.” p. 256
Hajek et al., 1997	Examined patients’ performance on 11 multifaceted stroke assessments. Determined… “cognitive functions are frequently affected in stroke patients, cognitive impairment needs to be more seriously considered when describing and/or predicting a patient’s level of independence.” p. 1331
Yakhkind et al., 2016	Reviews current evidence and standard of care for MiS patients. “A majority of patients with ischemic stroke present with mild deficits for which aggressive management is not often pursued. Comprehensive work-up and appropriate intervention for minor strokes and transient ischemic attacks (TIAs) point toward better patient outcomes, lower costs, and fewer cases of disability. Imaging is a key modality to guide treatment and predict stroke recurrence.” p. 86
Balasooriya-Smeekens et al., 2016	“Stroke and transient ischemic attack (TIA) survivors reported residual impairments that for many had impact on work. Most impairments were ‘invisible’, including fatigue, problems with concentration, memory and personality changes. …Despite having been able to return to work after a stroke, people may still experience difficulties in staying in work and risking losing their job.”
Adamit et al., 2015	Prospective cohort study of interrelationships among cognition, participation and quality of life (QoL) among MiS survivors. Participants experienced neurocognitive impairments negatively impacting participation and QoL. Health care systems and rehabilitation programs neglect the rehabilitation needs of MiS patients as they are seemingly independent with basic daily tasks. MiS has long-term effects impacting patients and their family members.
Barbay et al., 2018	Systematic review and meta-analysis of prevalence in post-stroke neurocognitive disorders (NCD) found 36% prevalence among MiS survivors. Argued that “Harmonization of stroke assessment and cognitive score thresholds is urgently needed to allow more accurate estimation of post-stroke NCD prevalence, especially mild post-stroke NCD.” P. 322
**Neurocognitive Function and VCI**	
de Haan et al., 2006	Cognitive and emotional outcomes are linked to: (a) focal damage causing selective impairments from gray matter dysfunction; (b) diffuse neuronal dysfunction compromising mental speed, memory, and executive functioning; and (c) outcomes being modulated by age, sex, premorbid level of functioning, and comorbidity. Modern neuropsychological assessment can facilitate patient classification, intervention selection, and creation of prognostic models.
Irimia et al., 2014	Reviews the state of the art in structural and connectomic neuroimaging targeting brain atrophy, alterations in morphometry, and inter-regional connectivity post injury. Discusses monitoring of clinical condition and evolution of status, including the potential for translational impact when coupled with neuropsychological measures.
Irimia and Van Horn, 2015b	Summarizes the use of functional magnetic resonance imaging, diffusion tensor imaging, positron emission tomography, magnetic resonance spectroscopy, and electroencephalography for studying TBI-related brain dysfunction and improving rehabilitation. Neurocognitive aspects discussed include consciousness, attention, memory, higher cognition, personality, and affect. Provides recommendations for future neuroimaging research.
Bullmore and Sporns, 2009	Highly influential review of complex brain networks in diverse experimental modalities (e.g., structural and functional MRI, diffusion tensor imaging, magnetoencephalography and electroencephalography).
Turken et al., 2008	Demonstrated correlation between Digit-Symbol performance and fractional anisotropy of white matter in bilateral parietal and temporal lobes and the left middle frontal gyrus. Assessed the effect of white matter damage on processing speed using voxel-based lesion-symptom mapping. Results indicated that cognitive processing speed is correlated with the structural integrity of white matter tracts associated with parietal and temporal cortices, left middle frontal gyrus, and the superior longitudinal fasciculus.
Gawryluk et al., 2014	Study employing 4T fMRI and an adapted Symbol Digit Modalities Test to identify white matter activation in either the corpus callosum or internal capsule in 88% of participants. “A key step in linking neuroimaging advances to the evaluation of clinical disorders is to examine whether WM activation can be detected at the individual level during clinical tests associated with WM function.”
Adleman et al., 2002	“… the first developmental fMRI investigation of the Stroop interference task, provides a template with which normal development and neurodevelopmental disorders of prefrontal cortex function can be assessed.”

**References**	**Summary Points**

**Neuroimaging and Neuroinformatics**	
Lim and Kang, 2015	Outlines the basic concepts of structural and functional connectivity, and the connectome. Explores current evidence regarding how stroke lesions cause changes in connectivity and network architecture parameters. Discusses clinical implications of perspectives on the connectome in relation to the cognitive and behavioral sequela of stroke. p. 256
Eckert, 2011	Neuroimaging morphometry study on neurobiological predictors of age-related changes in processing speed. Declines in specific neural systems compromise processing speed. “Future studies of processing speed – dependent neural systems will be important for identifying the etiologies for processing speed change and the development of interventions that mitigate gradual age-related declines in cognitive functioning and enhance healthy cognitive aging.” p. 25
**MiS-VCI rehabilitation and links to neuroanatomic, structural, and functional connectomics research**	
Park and Friston, 2013	Context-sensitive integration during cognition tasks entails a divergence between structural and functional networks. Function-structure mapping is crucial for understanding the nature of brain networks. The emergence of dynamic functional networks from static structural connections calls for a formal (computational) approach to neuronal information processing that may resolve this dialectic between structure and function. p. 579
Petersen and Sporns, 2015	“…network science offers a theoretical framework for approaching brain structure and function as a multi-scale system comprised of networks of neurons, circuits, nuclei, cortical areas and systems of areas. This paper views large-scale networks at the level of areas and systems, mostly based on data from human neuroimaging, and how this view of network structure and function has begun to illuminate our understanding of the biological basis of cognitive architectures.” p. 207
Kuceyeski et al., 2016	Demonstrated that “measures of baseline connectome disruption, acquired using only routinely collected MRI scans, can predict 6-month post-stroke outcomes in various functional domains including cognition, motor function and daily activities.” p. 2587
Liew et al., 2020	“… outlines the efforts taken by the ENIGMA Stroke Recovery working group to develop neuroinformatics protocols and methods to manage multisite stroke brain magnetic resonance imaging, behavioral and demographics data. Specifically, the processes for scalable data intake and preprocessing, multisite data harmonization, and large-scale stroke lesion analysis are described, and challenges unique to this type of big data collaboration in stroke research are discussed. Finally, future directions and limitations, as well as recommendations for improved data harmonization through prospective data collection and data management, are provided.” p. 129
Lopes et al., 2021	“Prediction accuracy was evaluated in four domains (memory, attention/executive, language and visuospatial functions) and compared with clinical data and other functional networks… A machine learning model based on the post-stroke cognitive impairment network can predict the long-term cognitive outcome after stroke.” p. E1167
**Knowledge translation approach to improving stroke care**	
Austin, 2021	“The mission of translational science is to bring predictivity and efficiency to the development and dissemination of interventions that improve human health… Reviews the origins of translational science and advances to this point.” p. 1629
Meyer, 2010	“Knowledge brokers are people or organizations that move knowledge around and create connections between researchers and their various audiences. This commentary reviews some of the literature on knowledge brokering and lays out some thoughts on how to analyze and theorize this practice.” p. 118
Chaudoir et al., 2013	Systematic review of structural, organizational, provider, patient, and innovation level measures affecting implementation of health innovations. “…discussion centers on strategies that researchers can utilize in order to identify, adapt, and improve extant measures for use in their own implementation research. In total, our literature review and resulting measures compendium increases the capacity of researchers to conceptualize and measure implementation-related constructs in their ongoing and future research.” p. 1

## Background

### Stroke Diagnosis and Classification

Currently, stroke is diagnosed based on neuroimaging and clinical examinations. Aberrant clinical examinations often reflect ischemia-associated fragmentation of neural networks and are highly correlated with abnormal findings on CT or MRI (Jadhav et al., 2020). More severe levels of stroke are typically accompanied by overt motor, sensory, and neurocognitive impairments. By contrast, MiS often involves a small ischemic lesion that may go undetected even when employing standard CT imaging. Detecting subtle deficits is further complicated by an upstream dearth of information concerning MiS-VCI. Lacking a consensus taxonomy for MiS, clinicians often rely on imprecise or inadequate categorizations of symptoms and sequelae. Such conceptualizations are frequently derived from routine neurological assessment protocols which quantify post-stroke severity and functional impairment but fall short of informing rehabilitation approaches (Hajek et al., 1997; Wolf and Rognstad, 2013). Importantly, many of these metrics quantify interrelationships between motor deficits and patients’ daily functioning by focusing on the ability to perform activities of daily living (ADL), bed mobility, transfers, and ambulation, rather than neurocognitive capacities (Wolf and Rognstad, 2013; Lee et al., 2015). Within this review, we use the overarching TREAT definition of MiS (Spokoyny et al., 2015) (i.e., NIHSS 0-5, and the absence of visual field deficits, aphasia, pronounced weakness, or other disabling deficits).

### Neurocognitive Function, Vascular Cognitive Impairment, and Functional Cognition

“Neurocognitive” refers to the interrelated domains of perceptual-motor function, language, social cognition, complex attention, executive function (EF), learning, and memory (Ganguli et al., 2011). VCI refers to the broad spectrum of neurocognitive impairment associated with vascular pathology, foregrounding working memory, PS, and EF as sensitive categorical impairment indicators (Hachinski et al., 2006). VCI is arguably a key defining characteristic of MiS and a robust predictor of successful participation in normal daily routines (Khatri et al., 2010; Spokoyny et al., 2015; Overdorp et al., 2016). Nevertheless, VCI is often overlooked in acute clinical settings despite multilevel implications for patients, health care systems, and society (Tellier and Rochette, 2009; Ovbiagele et al., 2013; Adamit et al., 2015). This disconnection stems from insufficient MiS diagnostic criteria, with underdeveloped characterizations impeding deficit identification and remediation (Fischer et al., 2010; Roberts et al., 2020). Even using the term *mild*, in this context, can inadequately represent the persistent obstacles within post-stroke daily functioning (Burns et al., 2018). Lastly, “functional cognition” relates to OT’s focus on “clients” and their capacity to perform essential tasks given the totality of their abilities, including their use of strategies, habits/routines, and contextual/environmental resources (Giles et al., 2017). Although functional cognition requires looking beyond discrete skills, we contend that initially employing a sound, generalized proxy for network dysfunction can increase access to more specific, comprehensive, and ecologically valid types of intervention currently missing from MiS care.

Neurorehabilitation research and practice are moving toward performance-based assessment tools, such as the Executive Function Performance Test (Baum et al., 2008), the Menu Task (Al-Heizan et al., 2020), and the Kettle Test (Hartman-Maeir et al., 2009). Yet, traditional instruments such as the Trail Making Test (Reitan, 1958), Symbol-Digit Modalities Test (SDMT) (Smith, 1973), and Stroop Color-Word Test (SCWT) (Golden and Freshwater, 2002) remain part of the gold standard for validating newer instruments. Despite newer tools achieving higher ecological validity by evaluating real-world task performance, their administration requirements often strain clinician capacities given time and environmental limitations inherent to practice, especially in fast-paced hospital settings.

In this review, we highlight the use of PS to detect compromised connectome integrity. We further explore how assessing VCI via the select, psychometrically robust instruments discussed herein can significantly improve early stroke care services. In particular, circumscribing and formalizing early MiS-VCI detection protocols can help identify subtle deficits that may be otherwise overlooked. Further, more uniform, sensitive, and specific assessments can potentiate greater access to subsequent performance-based OT evaluations emphasizing functional cognition.

#### Mild Stroke-Vascular Cognitive Impairment Underdetection

Uncertainty surrounding the concept of MiS-VCI derives in part from inadequate in-place assessment protocols (Roberts et al., 2020). This shortcoming is troubling given that VCI is common among MiS survivors (Chung et al., 2013; Spokoyny et al., 2015; Overdorp et al., 2016) and involves subtle—yet persistent—deficits in working memory and EF. Importantly, PS (i.e., “the rapidity with which a patient processes simple or routine information without making errors of either omission or commission,” Weiss et al., 2010) can sensitively detect MiS-VCI. Improper or inadequate computation within neural networks can significantly impact patients’ ability to undertake daily life tasks, especially when required speed, complexity, and duration amount to high cognitive loads (Khatri et al., 2010; Wolf and Rognstad, 2013; Spokoyny et al., 2015). To offset such potential issues, we wish to extend the scope of care available to the underserved MiS population.

Building on existing literature, we aim to advance MiS-VCI knowledge translation targeting clinical research. To this end, we discuss MiS-relevant scope, implications, and opportunities afforded by structural neuroimaging and connectomics research, though without deeply exploring overarching MiS and VCI associations that are thoroughly reviewed elsewhere (Brodaty et al., 2010; Spitzer et al., 2011; Li et al., 2012; Wolf and Rognstad, 2013; Spokoyny et al., 2015; Sensenbrenner et al., 2020). More concretely, we propose a novel course of action informed by neuroimaging, connectomics, network science, and OT that quantifies covert VCI and highlights the mechanistic link to connectopathies. Our central thesis holds that, in light of technological innovation and advances in neuroimaging and neuroinformatics, PS has emerged as an underutilized—yet essential—indicator of compromised neural network integrity, and thus as a viable MiS-VCI biomarker.

Acute stroke screening protocols lack the ability to consistently detect subtle MiS-VCI. Not having sensitive indicators of potential VCI can potentiate patients being ill-prepared to resume full participation in their daily routine. Being unaware of possible neurocognitive deficits, MiS survivors may prematurely recommence complex tasks such as driving, employment, and caregiving. Therefore, we support using robust capacity indicators to quantify potential discrepancies between pre- and post-stroke levels of functioning.

Mild stroke survivors experience less sensorimotor and VCI than those with moderate-to-severe strokes (Tellier and Rochette, 2009). Yet, they have similar rates of disability (19%, Cucchiara et al., 2019) as all stroke severity classifications as a whole (22%, Mu et al., 2017). Current methodological approaches for understanding and quantifying the nuances of MiS-VCI cannot explain the mechanisms that underlie these findings. Therefore, a critical question is: What is the source of MiS survivors’ documented disability?

Even subtle VCI can manifest a profound functional disconnection between past and present capacities when individuals undertake complex tasks (Stephens et al., 2005). In practice, there are widely accepted measures of motor function such as the Fugl-Meyer assessment (Fugl-Meyer et al., 1975) and the Action Research Arm Test (Lyle, 1981; Santisteban et al., 2016). Yet, uncertainty regarding the measurement and classification of MiS-VCI persists. In this context, focusing on MiS-VCI may yield promising etiological insights (de Haan et al., 2006) via ecologically valid perspectives on daily task proficiency (Stephens et al., 2005).

Profound VCI unquestionably contributes to stroke-associated disability (Spitzer et al., 2011; Wolf and Rognstad, 2013; Sensenbrenner et al., 2020). Yet, there is an absence of research examining the influence of PS thresholds on the likelihood and severity of post-stroke disability. This knowledge gap stems from the widespread practice of classifying stroke by heavily weighting the degree of motor impairment, leading to estimates that 88% of stroke cases acquire notable motor deficiencies, with 71% failing to regain motor function within 6 months of onset (Bonita and Beaglehole, 1988). Among those with TREAT-defined MiS, 29-35.6% reportedly experience residual deficits (Spokoyny et al., 2015). Regarding MiS-VCI, focusing on the degradation of network efficiency to better understand post-stroke disability reveals a path to move beyond the circumscribed utility of primarily motor-driven assessments.

#### Impact of Underdetection

Stroke survivors with less pronounced impairments are more likely to resume participation in complex activities that offer little margin for error (Hofgren et al., 2007). This tendency may help explain how MiS survivors that lack overt deficits ultimately file for disability at relatively similar rates to those with higher degrees of documented impairment (Mu et al., 2017). To a potentially detrimental degree, decisions regarding activity participation are likely scaled according to patients’ perceived residual capacity. This perspective derives from individuals’ reflections on their lived experience, combined with the degree to which healthcare providers have endorsed their overarching capacities. Though predominantly missing from discussions in contemporary practice, PS provides a sound framework for assessing the integrity of neurocognitive operations in a way that can bridge the gap between actual and perceived abilities. As detailed in subsequent sections, PS can provide a valuable, quantifiable proxy for neurocognitive function. Moreover, it can drive cutting-edge knowledge translation efforts targeting stroke severity characterization and recovery prediction.

Stroke survivors discharging from the acute hospital setting with unrecognized MiS-VCI face considerable challenges in resuming safe and independent daily routine participation (Balasooriya-Smeekens et al., 2016; Camicia et al., 2016). Unfortunately, there is a dearth of literature coalescing clinical evidence to compel early, highly sensitive neurocognitive assessment that could prompt referrals to essential outpatient therapy services following hospitalization. Without improved, neurocognitively focused early stroke care screening protocols, MiS will remain inconsistently detected, incompletely understood, and MiS survivors will continue to struggle in silence (Barbay et al., 2018).

Despite unequivocal evidence linking functional cognition to successful ADL performance (Chung et al., 2013; Fride et al., 2015; Overdorp et al., 2016), widely used stroke assessments lack the sensitivity to detect subtle VCI (Wolf and Rognstad, 2013; Yakhkind et al., 2016; Burns et al., 2018). Concerns about the limited scope of diagnostic instruments are compounded by the frequent suspicion that some MiS patients are incorrectly perceived as lacking deficits (Yakhkind et al., 2016). As a result, MiS patients are often discharged with unrecognized VCI after brief hospital admissions (Balasooriya-Smeekens et al., 2016; Camicia et al., 2016), and are likely to experience threshold-dependent VCI.

Broadly conceived, threshold-dependent VCI involves diminished neurocognitive functioning linked to activity-dependent increases in cognitive load. Daily living involves fluctuating requirements in speed, complexity, and duration inherent to activity engagement. PS metrics can quantify maladaptive responses to variable, multifocal task demands (Leavitt et al., 2011). Thus, increased cognitive load, reflecting greater requisite mental effort during task performance (Calvillo and Irimia, 2020), is linked to diminished task performance quality (Hajek et al., 1997; Fischer et al., 2010). Clinical stroke protocols can benefit from incorporating elements informed by responses to cognitive load and likely individual-specific performance variability.

### Knowledge Translation

Entrenched disconnections between research discovery and carry over into clinical practice reinforce a longstanding barrier to mutually beneficial exchanges of ideas (Austin, 2021). Previous efforts targeting top-down, direct-translation of knowledge to end-users have attempted to rectify this issue, with recent trends favoring intricate, multilevel stakeholder collaborations. Within a knowledge translation framework, OT is highly qualified to incorporate innovative approaches into multidisciplinary care collaboratively. Such ends can be accomplished by OT occasionally acting as the knowledge broker, a designation characterizing persons that “facilitate the creation, sharing, and use of knowledge” (Meyer, 2010).

Given OT’s expertise in addressing functional cognition within dynamic, real-world contexts, practitioners can facilitate valuable MiS-VCI research collaborations mobilizing neuroimaging and connectomics-based knowledge. In particular, they can lend insight into the multifaceted nature of patients’ impairments, an essential component of innovative predictive models, patient care delivery, and communications with various stakeholders. Among allied health professions in acute care settings, OT is the only one to have demonstrated a statistically significant association between healthcare spending and lower readmission rates (Rogers et al., 2017). In line with NIH’s emphasis on innovation and developing evidence-based treatment, OT’s established expertise, in-place representation within acute care teams, and cost-effective services provide strong support for its potential to broker knowledge toward those ends effectively (Chaudoir et al., 2013).

### Neuroimaging-Based Perspectives and Future Mild Stroke Care

#### Neuroimaging and Neuroinformatics for Mild Stroke Care

Neuroimaging technologies have added unprecedented scope and precision to our understanding of neurological disorders. Unfortunately, current advances in neuroimaging and neuroinformatics outpace translational efforts targeting MiS care. Given the symbiotic nature of translational research and clinical practice, such delays have hindered ongoing innovation in both domains. Although evidence-based protocols guiding early stroke care have improved recovery trajectories, common metrics quantifying MiS characteristics do not reflect recent insights into complex neural processes underlying observable clinical deficits. Instead, contemporary early stroke clinical assessments inordinately focus on motor capacity over cognitive function (Bonita and Beaglehole, 1988; Hodics et al., 2012), endorsing an underdeveloped representation of MiS sequelae.

Connectomics is the field of study concerned with the systematic mapping of neural connections. It provides a unique framework for analyzing neural network disruptions by combining graph theory methodology with neuroimaging (Irimia et al., 2014). Specifically, by conceptualizing brain regions as nodes with structural or functional properties connected by white matter (WM) fibers, connectomics can bridge the gap between mapping neural networks and understanding their functions (Bullmore and Sporns, 2009). Neural network analysis involves focusing on both functional and structural connectivity (Irimia and Van Horn, 2015b). Employing diffusion tensor imaging (DTI) and diffusion spectrum imaging, *structural* connectomics uses spatial topography data to construct a physical map of neural activity and infer WM anatomic organization along with neurological insult-derived connectivity damage (Irimia et al., 2014).

Using functional MRI (fMRI) (Irimia and Van Horn, 2015b), magnetoencephalography (MEG) (Irimia and Van Horn, 2015a), and electroencephalography (EEG) (Lima et al., 2006; Irimia and Van Horn, 2015a) *functional* connectomics involves the study of neural network activity via: (a) temporal dynamics; (b) maps representing information exchange between nodes and modules; (c) resting states; and (d) responses to exogenous stimuli (Halgren et al., 2011). Such studies facilitate the measurement of network integrity following neurological insults, including stroke. Additionally, brain activity can be further understood mechanistically through activation patterns identified within the functional connectome. This lens can be useful when cognitive abilities, behavior, or network PS modulate neural responses (Craddock et al., 2015).

Ischemic insults can compromise information processing within and across neural networks. In worst-case scenarios, this ability is lost. In more moderate cases, neuroplasticity can facilitate the preservation of intended operational outputs in a lesioned network. Importantly, this type of recovery has been studied more thoroughly in sensorimotor than neurocognitive domains (Gray et al., 1989; Wang et al., 2010; Lee et al., 2015). With neurocognitive demands, however, negative consequences of ischemic insults may manifest as sub-optimal neural processing, producing operations that are detrimental to the desired outcome or are inadequately timed. Such imprecision gives rise to alternative neural pathway use during network activation sequences to meet specific PS demands (Honey and Sporns, 2008; Wang et al., 2010). By integrating connectomics insights into practice and knowledge translation initiatives, rehabilitation professionals can better identify, isolate, and subsequently target alternative pathways within patient-tailored therapeutic activities.

Consistent with previously discussed trends comparing VCI across stroke severity levels, the ubiquity of WM damage and compromised neurocognitive function is well-documented among severe cases (Zinn et al., 2007), and significantly less so among those with lower degrees of functional impairment (Wolf and Rognstad, 2013). Using innovative PS measures can more sensitively detect MiS-VCI than standard assessment protocols. The following two sections review evidence linking common processing efficiency metrics to emerging insights from connectomics. Such evidence, we propose, can aid the formulation of novel, concrete guidelines for utilizing such knowledge in clinical rehabilitation practice and knowledge translation endeavors.

#### Insights From Structural Connectomics

Previous studies have highlighted relationships between PS and the modulation of WM connections. For example, Turken et al. (2008) demonstrated that damage to the structural integrity of WM axons correlates with diminished performance in PS tasks. Specifically, posterior parietal lesions often lead to compromised PS and associated changes in the integrity of water diffusion anisotropy along WM fibers. Additional evidence suggests that brain activity recruiting temporo-occipital WM structures, such as the inferior longitudinal fasciculi, also modulates PS (Turken et al., 2008). There is robust support for the use of well-studied, psychometrically sound neurocognitive assessments targeting processing efficiency. For example, the SDMT evaluates PS and efficiency and is sensitive to WM disruptions (Gawryluk et al., 2014), whereas the SCWT includes a useful event-related cognitive task that can measure PS sensitively even in the presence of subtle VCI (Adleman et al., 2002).

DTI techniques like WM tractography facilitate the modeling and visualization of neural pathways (Basser and Pierpaoli, 2011). This method has been used to employ compromised processing efficiency as a metric for impairment magnitude across various neurocognitive domains. Action potentials propagating along WM axons enable information exchange across neural networks, often by synchronizing the firing of neuronal populations (Assaf and Pasternak, 2008; Turken et al., 2008; Basser and Pierpaoli, 2011). Thus, WM insults can substantially alter information processing across network nodes, disrupting neurocognitive capacities (Assaf and Pasternak, 2008; Turken et al., 2008; Basser and Pierpaoli, 2011). At the microscale, such insults often involve dendritic or synaptic loss and inflammation modulated by microglia, both phenomena being able to interfere with local network function (Lim and Kang, 2015).

In addition to applications for MiS-VCI, given DTI’s ability to identify subtle WM abnormalities, it can assist in predicting functional outcomes in other cases involving less severe neurological insults. In particular, there is support for this technology addressing WM changes associated with cerebral hemorrhages and mild traumatic brain injury (Irimia et al., 2014), as well as those linked to the neuro-invasion potential of SARS-CoV-2 during the recovery from COVID-19 (Lu et al., 2020). Importantly, standard structural MRI cannot always detect WM changes (Lim and Helpern, 2002; Assaf and Pasternak, 2008). Thus, DTI can be leveraged to assist the diagnosis and classification of MiS, and help researchers elucidate the relationship between structural WM damage and MiS-VCI. Additionally, because DTI-based network analysis can provide quantitative measures of brain dysfunction, this strategy can complement in-place behavioral diagnostic methods. For these reasons, we propose that insights obtained by combining quantitative descriptions of structural network integrity and insult location can have utility for improving diagnostic accuracy while facilitating MiS characterization and targeted treatment-approach development.

#### Insights From Functional Connectomics

In its own right, functional connectivity can substantially assist MiS diagnosis and classification. Research suggests that the brain can be modeled as a small-world network with extensive local clustering, which facilitates efficient information processing (Lim and Kang, 2015). Global communication, by contrast, relies on long-range connections between network nodes. This ranged approach enables further information processing and integration across neuroanatomically or functionally distinct regions. The extent of network impairment following stroke is highly dependent upon lesion location and can alter both local and global network efficiencies (Wang et al., 2010; Lim and Kang, 2015). For example, Wang et al. (2010) studied patients with focal subcortical motor pathway damage and found that, compared to healthy volunteers, motor execution networks exhibited lower efficiency of local information transfer between homotopic brain network regions. Similarly, reductions in global network efficiency due to nodal damage have been demonstrated by both computational models and MRI studies (Honey and Sporns, 2008; Alstott et al., 2009; Wang et al., 2010). Therefore, interpretations and clinical applications of connectomic measures require careful consideration.

While stroke may compromise local network efficiency, distal network modules can assume the roles of injured regions in response to rehabilitation (Biernaskie et al., 2005; Nomura et al., 2010; Grefkes and Fink, 2011). Given structural redundancies within the brain, these networks can be recruited during recovery from stroke, potentiating functional improvement. Whereas local network efficiency can decrease following stroke, there can also be simultaneous, compensatory functional connectivity increases throughout the brain. Compensation may involve rerouting information between nodes using indirectly connected uninjured regions, increasing the amount of neural processing commensurate with task demands.

Researchers can harness functional connectomics to enhance investigations of cortical information exchange and network integrity since connectomic analysis can sensitively detect subtle MiS-VCI. Furthermore, functional connectivity methods can facilitate the investigation of task-related, inter-regional coupling (Wang et al., 2010), phenomena that are essential considerations in conceptualizing VCI. Incorporating these insights into MiS care can bolster clinical assessment methods and improve the detection of subtle network inefficiencies associated with context-driven impairment.

### PS: A Proxy for Complex Neurocognitive Function

To assess network processing efficiency and quantify the adequacy of mental operations indelible to neurocognitive function, clinical assessments should account for patient-specific factors that facilitate or hinder performance. Replicating stressors that fuel context-driven impairment can be impractical in acute care settings. In this light, there is utility in identifying a sound proxy to screen core neurocognitive capacities. One crucial factor that varies in response to context-driven cognitive demands is PS. Task-specific demands governing speed, complexity, and duration parameters influence the degree of perceived difficulty associated with any given neurocognitive operation.

Distractions and unexpected changes in the scope of a given task can negatively impact processing efficiency by increasing the cognitive load. Such challenges increase demands upon working memory and EF. Given the nuanced nature of daily living, difficulties may still ensue after reducing competing stimuli and prioritizing task resources. Within the literature, there are examples linking compromised PS to deficient saccadic eye movements and oculomotor impairment to neurocognitive dysfunction (Barnett and Singman, 2015). Similarly, the SCWT and the SDMT are gold-standard assessments of PS (Smith, 1973; Golden and Freshwater, 2002). Importantly, results from these instruments derive from large normative statistical samples and allow subject stratification according to age and educational attainment peers. These tests have also been used to measure neurocognitive function under heightened cognitive load (Siegle et al., 2008) and detect deficits ranging from subtle to profound impairment. In conclusion, using PS as a proxy to assess connectomic integrity and subsequent neurocognitive capacities provides a central organizing principle to inform MiS-VCI detection protocols, drive knowledge translation efforts, and refine treatment approaches.

## Discussion

### Clinical Applications and Considerations

Building on the Human Connectome Project’s body of knowledge, current conceptualizations of intact network processing (Sporns et al., 2005), generalized linear models of neurocognitive function (Park and Friston, 2013; Petersen and Sporns, 2015), and studies of damaged network integrity due to focal or diffuse injuries (Lim and Kang, 2015), the potential benefit of incorporating connectomic methods into MiS care protocols is compelling. Work toward this end involves creating functional connectivity models, plus calculating network clustering coefficients and characteristic path lengths (Bullmore and Sporns, 2009). Thus, improved MiS-VCI detection could involve quantifying neural network efficiency or the extent of network reorganization following injury. Such strategies offer the potential to stratify MiS presentations with substantial sensitivity and specificity based on the severity of functional network impairments. This approach has already been demonstrated in proof-of-concept studies involving moderate-to-severe stroke (Wang et al., 2010), suggesting that extending this method to more accurately characterize MiS-VCI should be a future research priority.

While clinical neuroimaging modalities critically inform medical management decisions following moderate and severe stroke (Wintermark et al., 2013), multimodal imaging and network theory research have substantially advanced our current understanding of structural and functional connectivity disturbances underlying MiS (Silasi and Murphy, 2014). Such progress has enabled clinicians and researchers to better characterize stroke lesions and their impact on structural and functional connectivity. In practice, neuroimaging localizes lesions topographically to construct a predictive framework for cognitive outcomes after stroke (Hope et al., 2013). However, most topographic models do not account for stroke severity (van Meer et al., 2012; Hope et al., 2013). Thus, aiming beyond topography-based lesion analysis (e.g., studying stroke-driven structural connectome alterations) can potentially help predict MiS-VCI incidence and identify associated biomarkers (Kuceyeski et al., 2016). To improve MiS care, clinician scientists, OT, and other allied health professions should acquire and incorporate findings from standard structural and multimodal imaging technologies. Employing aggregate, atlas-informed (Liew et al., 2020) neuroimaging biomarkers can aid MiS classification, reducing uncertainty stemming from absent consensus on best practices governing detection and diagnosis. Although MiS and brain network heterogeneity may be limiting factors in this respect, incorporating knowledge from VCI-associated deficit atlases can refine clinical evaluations (Wang et al., 2010). Insights from these data-driven approaches may inform the development and selection of MiS-VCI rehabilitation approaches rooted in daily living contexts. However, what is the best way to employ multimodal approaches in quantifying network efficiency and PS while also deriving more sensitive and specific MiS-VCI characterizations? One process could involve the creation or refinement of resting-state fMRI atlases and mapping MiS-associated functional network impairments. This type of development would be akin to MR fingerprinting techniques (Ma et al., 2013) and the Virtual Brain Project, where neuroinformatics drives personalized medical care (Falcon et al., 2016).

Advances in network theory and neuroimaging applications can help clinicians develop innovative, evidence-based, early MiS care. For example, the literature supports the utility of studying compromised network efficiency linked to damaged neuroanatomical structures (Honey and Sporns, 2008). Due to stroke-related increases in average neural network path lengths, information relayed across these structures takes longer to reach intended destinations. This insult-derived change supports screening for MiS-VCI using PS metrics (Turken et al., 2008; Eckert, 2011), as PS is a key variable predicting overall cognition-based stroke outcomes (Su et al., 2015).

As previously discussed, VCI can be reliably measured at the bedside by OT using robust instruments like the SDMT and SCWT (Smith, 1973; Golden and Freshwater, 2002). Additionally, compromised PS can disrupt essential visual-motor skills such as pursuits and saccades. These foundational visual-motor abilities are sensitive to mild cortical insults and can reflect the integrity of core neurocognitive capacities at risk for context-driven impairment (Barnett and Singman, 2015). Therefore, healthcare providers should integrate this information into early stroke care protocols to increase MiS-VCI detection and subsequent access to services (e.g., outpatient OT targeting functional cognition and post-stroke quality of life).

Looking beyond applications within *acute* MiS care, neuroimaging advances could inform more effective functional cognition protocols for *chronic* insult recovery. Historically, clinicians have relied heavily upon assessment batteries or subjective measures to determine intervention efficacy (Hajek et al., 1997). However, research suggests that technology such as DTI can be employed to bolster predictions regarding motor responses to restorative stroke therapies (Cassidy et al., 2018). The validity of such approaches targeting MiS-VCI would be further strengthened by in-depth analyses of network disruptions within specific regions of interest, quantified by measures like connectivity strength (Sporns and Zwi, 2004; Park and Friston, 2013).

### Future Directions

In addition to improving detection and service provision for stroke patients, emerging practices involving multimodal neuroimaging are being employed to model and predict clinical outcomes. For example, recent studies have successfully predicted aspects of post-stroke cognitive recovery by examining DTI-based connectivity measures and employing machine learning analysis of resting-state network fMRI data (Aben et al., 2021; Lopes et al., 2021). Unfortunately, these studies focus on long-term timelines, using measures acquired 6 months after stroke to predict function at 3-years, or 5 weeks post onset being used to predict 1-year status. We contend that, while such studies are important to advance our overarching understanding of stroke, we must also look pointedly at immediately pressing issues. Specifically, steps should be promptly taken to improve triaging and care for individuals experiencing MiS.

The clinical and neuroimaging communities should undertake sustained knowledge translation efforts to develop theory-driven, practice-based treatment models addressing the paucity of knowledge regarding MiS-VCI. As exemplified in the examples below, integrating neuroimaging protocols with clinical rehabilitation insights can enhance our understanding of this understudied condition. For instance, bolstering functional cognition assessments to consider network strain under high cognitive loads can improve MiS-VCI characterization. Also, positron emission tomography (PET) or MR spectroscopy (MRS) can potentiate improved post-stroke functional mapping and pharmacological targeting of performance-specific neurotransmitters and blood-borne molecules (Schlosser, 2000). Further, evidence supports that incorporating neuroimaging insights into pharmaceutical development protocols can improve early post-stroke medication effectiveness (Faingold and Blumenfeld, 2015). One potentially beneficial general consideration regarding MRI segmentation, as the accuracy of its scheme relies on the ability to differentiate between tissue classes (Madhukumar and Santhiyakumari, 2015) is that the use of fuzzy preprocessors can account for tissue geometry and reduce computational load (Versaci et al., 2015). Lastly, promising MEG/EEG mapping research supports that *temporal* network patterns reflect resource shifts pre, during, and post task performance (Irimia and Van Horn, 2015b). Studies such as these, examined within large samples, could elucidate high-frequency, time-dependent characteristics of network activity, against which deficient MiS-VCI dynamics can be compared. However, considerable work remains to clarify interrelationships between MiS-driven electrophysiological changes during neural activity and links to clinical parameters.

## Conclusion

Subtle stroke-associated neurocognitive impairment is an under-examined condition that impacts patients, health care systems, and society overall (Sun et al., 2014). Though two-thirds of stroke survivors experience minor deficits (Yaghi et al., 2017), and the emerging recognition that VCI is a potential hallmark of MiS (Lim and Kang, 2015), conventional assessments often mischaracterize or inadequately detect MiS. Existing diagnostic and rehabilitation paradigms would benefit from incorporating current perspectives from neuroimaging and connectomics. Such inclusion would illustrate research and practice symbiosis, via knowledge translation efforts, targeting improved MiS-VCI construct clarification and clinical service provision.

Incorporating connectomics and network theory into existing clinical perspectives may require further innovation targeting the acquisition, analysis, and interpretation of neuroimaging data. Nevertheless, structural and functional neuroimaging methods offer unique opportunities for scientific discovery and clinical advances in MiS care whether employed individually or together (Silasi and Murphy, 2014). Available literature supports two key approaches for developing and refining current perspectives: (a) quantify interrelationships between MiS-VCI and network dysfunction via existing neurocognitive assessments; and (b) atlas MiS-derived neural damage to bolster interdependence of MiS research and clinical practice (Liew et al., 2020). These and other strategic knowledge-translation efforts should be pursued in the understudied yet epidemiologically significant area of MiS-VCI.

Finally, considering the MiS-survivor experience, returning to prior routines can be fraught with challenges. Performing complex activities with neurocognitive deficits can be complicated by a narrow margin of error separating success from failure. Consider the prospect of resuming employment with undiagnosed MiS-VCI. A VCI-fueled unsuccessful return to work scenario is easy to envision without access to sensitive assessment and treatment. Despite legal protections available through the Americans with Disabilities Act (n.d.), there is still an inordinate risk for performance-based termination if MiS-VCI has not been identified.

Every day, lapses in judgment and substandard quality of work drives job loss among neurologically intact individuals. In this light, how are MiS-VCI survivors likely to fare given an increased probability of substandard complex task performance in conjunction with potential overestimations of their capacities? Without sensitive MiS-VCI assessments and established access to ecologically valid functional cognition interventions, MiS survivors are often ill-equipped to fully participate in the context of daily living. They are also likely to struggle in negotiating the impact of multifaceted functional disconnections from their baseline capacities. Such deficiencies can be life-changing, significantly altering their ability to perform other complex tasks such as driving and caregiving. In response to such potentialities, clinicians and researchers should proactively infuse cutting-edge neuroimaging technology, neuroinformatics, and connectomics perspectives into rehabilitative approaches to positively influence MiS survivors’ neurocognitive recovery trajectories.

## Author Contributions

All authors listed have made a substantial, direct, and intellectual contribution to the work, and approved it for publication.

## Conflict of Interest

The authors declare that the research was conducted in the absence of any commercial or financial relationships that could be construed as a potential conflict of interest.

## Publisher’s Note

All claims expressed in this article are solely those of the authors and do not necessarily represent those of their affiliated organizations, or those of the publisher, the editors and the reviewers. Any product that may be evaluated in this article, or claim that may be made by its manufacturer, is not guaranteed or endorsed by the publisher.
